# Domains of Miswak practice among Malaysian Dental Educators: insights from a focus group discussion

**DOI:** 10.1590/1807-3107bor-2026.vol40.071

**Published:** 2026-08-24

**Authors:** Muhd Firdaus Che Musa, Noorhazayti Ab Halim, Farah Natashah Mohd, Widya Lestari, Galvin Sim Siang Lin, Mohamad Shafiq Mohd Ibrahim

**Affiliations:** (a) International Islamic University Malaysia, Kulliyyah of Dentistry, Department of Paediatric Dentistry & Dental Public Health, Pahang, Malaysia.; (b) International Islamic University Malaysia, Kulliyyah of Dentistry, Department of Restorative, Pahang, Malaysia.; (c) International Islamic University Malaysia, Kulliyyah of Dentistry, Department of Fundamental Dental and Medical Sciences, Pahang, Malaysia; (d) International Islamic University Malaysia, Kulliyyah of Dentistry, Department of Restorative Dentistry, Pahang, Malaysia.

**Keywords:** Education, Dental, Qualitative Research

## Abstract

This study aimed to explore dental educators’ perceptions of the domains influencing the practice of miswak among dental educators. Dental educators from the International Islamic University Malaysia, representing diverse socio-religious backgrounds, participated in focus group discussions (FGDs) guided by a pre-tested topic framework. The discussions were audio-recorded, transcribed, and analyzed using the framework analysis approach. A total of 11 dental educators participated in four FGD sessions conducted. The analysis identified four interrelated domains influencing miswak practice: self-care, conventional teaching, research evidence, and political or policy context. Participants described miswak primarily as a culturally and religiously embedded oral hygiene practice, while also highlighting uncertainties regarding its integration into the dental education due to limited curriculum guidance and perceived gaps in evidence dissemination. Some participants argued that their Western-oriented dental training also influenced how miswak is perceived within the education system. Overall, the findings suggest that educators’ education and training background, together with their levels of knowledge and awareness, shape perceptions of miswak and influence its acceptance within dental education and practice. Further research may help inform educational guidance to support the appropriate integration of Miswak as a complementary oral hygiene practice.

## Introduction

Miswak (*Salvadora persica*), a traditional chewing stick used for oral hygiene and derived from natural resources, holds significant cultural and religious importance, particularly within Muslim communities.^1,2^ Its use dates back centuries and is often tied to religious practices.^3^ Beyond its symbolic value, scientific research has consistently documented its oral health benefits, ranging from plaque reduction to antimicrobial activity.^1^ Despite its longstanding history and well-documented oral health benefits,^4^ the incorporation of miswak into formal dental education and clinical practice remains sporadic. In many institutions, its use is acknowledged but not systematically taught, largely due to variations in dental educators’ knowledge, awareness, and attitudes toward the practice.^5^ This inconsistency has hindered its wider acceptance and routine incorporation into curricula and clinical protocols.

There is robust evidence supporting miswak's efficacy, and several systematic reviews and meta-analyses have confirmed the clinical efficacy of miswak in reducing dental plaque and gingivitis.^6-9^ In several instances, its performance has been found to be comparable to that of conventional toothbrushes, and in adjunctive use, it may even offer superior benefits.^8-11^ Nevertheless, concerns persist among educators over the lack of standardized protocols for its use and methodological inconsistencies across existing studies.^5,8,12^ The absence of clear, evidence-based guidelines creates uncertainty and presents a challenge for integrating miswak into teaching and practice confidently.

The limited dissemination of high-quality research findings further compounds this skepticism. Despite strong evidence supporting miswak's clinical value, skepticism persists among dental educators due to the limited dissemination of research findings and a lack of clear, evidence-based guidelines for implementation in the curriculum.^5,13^ Educators often report low to moderate levels of knowledge and awareness about miswak, and their views are heavily influenced by their cultural, religious, and educational backgrounds.^5^ Commonly cited barriers include a perceived insufficiency of rigorous evidence, a lack of institutional or policy-level support, and tensions between traditional practices and dominant Western-centric models of dental education.^5,13^

Moreover, qualitative studies conducted in Malaysia highlighted the localized and culturally nuanced understanding of miswak among dental educators.^5^ In this regard, the cultural and religious context often influences acceptance, sometimes creating friction within academic institutions where Western scientific standards dominate.^13,14^ Despite the high quality of clinical evidence supporting miswak, the limited scope of research on dental educators’ perspectives, often constrained by small, context-specific samples,^5^ suggesting a pressing need for more diverse and expansive studies. Bridging the divide between traditional oral hygiene practices and modern dental education will require multi-faceted strategies: developing culturally sensitive curricular frameworks, establishing standardized protocols for teaching and clinical use, and fostering policy-level support.^5,6,9^ Such efforts could legitimize miswak within both local and international educational settings, preserving cultural heritage while aligning it with evidence-based practice.

In light of these gaps, further research is needed to better understand how traditional oral hygiene practices such as miswak are perceived within contemporary dental education. While existing studies have documented its clinical benefits, limited research has explored how dental educators interpret and engage with miswak within academic and clinical contexts. Understanding these perspectives is important for identifying potential barriers and opportunities for integrating culturally significant oral health practices into evidence-based dental education. This study therefore aimed to explore dental educators’ perceptions of the domains influencing miswak practice within academic and clinical settings using focus group discussions.

## Methods

### Study design

A qualitative approach using focus group discussions (FGDs) was employed to explore dental educators’ perceptions of miswak practice. This method was chosen for its ability to capture rich and interactive data, as well as allowing participants to build on each other's perspectives. The study was conducted at Kulliyyah of Dentistry, International Islamic University Malaysia (IIUM).

Ethical approval was obtained from the IIUM Research Ethics Committee, under reference number IREC 771. The study was conducted in accordance with the World Medical Association Declaration of Helsinki. Written informed consent was obtained from all participants before data collection, including permission for audio-recording. Participants were assured of their confidentiality, the voluntary nature of their participation, and their right to withdraw at any stage without penalty. This qualitative study was reported following the Consolidated Criteria for Reporting Qualitative Research (COREQ) checklist to ensure methodological rigor and transparency.

### Study population

A total of 11 dental educators participated in the study. To obtain diverse perspectives, purposive sampling was used to ensure representation from different socio-cultural and religious backgrounds. Participants were stratified into four groups to facilitate the open discussions: Group 1 (FG1) comprised local Muslim educators; Group 2 (FG2), local non-Muslim educators; Group 3 (FG3), international Muslim educators; and Group 4 (FG4), international non-Muslim educators. This grouping was intended to reduce potential discomfort in discussing culturally or religiously sensitive topics. All participants held academic positions in dentistry and had experience in either clinical teaching, didactic teaching, or both. Demographic information, such as gender and country of origin (local or international), was recorded to contextualise the findings.

The sample size was considered appropriate for qualitative inquiry, where the aim is to achieve depth of understanding rather than statistical generalisation. The inclusion of participants from diverse socio-cultural and professional backgrounds, together with the achievement of thematic saturation after four focus group discussions, supports the adequacy of the sample.

A total of 11 dental educators participated in the study. To obtain diverse perspectives, purposive sampling was used to ensure representation from different socio-cultural and religious backgrounds. Participants were stratified into four groups to facilitate the open discussions: Group 1 (FG1) comprised local Muslim educators; Group 2 (FG2) consisted of local non-Muslim educators; Group 3 (FG3) involved international Muslim educators; while Group 4 (FG4) included international non-Muslim educators. This grouping was intended to reduce potential discomfort in discussing culturally or religiously sensitive topics. All participants held academic positions in dentistry and had experience in either clinical teaching, didactic teaching, or both. Demographic information, such as gender and country of origin (local or international), was recorded to contextualize the findings.

The sample size was considered appropriate for qualitative inquiry, where the aim is to achieve depth of understanding rather than statistical generalization. The inclusion of participants from diverse socio-cultural and professional backgrounds, together with the achievement of thematic saturation after four focus group discussions, supports the adequacy of the sample.

### Topic guide

The focus group discussions (FGDs) followed a structured topic guide that covered four main areas: a) individuals and their roles within the dental workforceb) benefits of miswak; c) domains of practice, including facilitators and barriers; and d) best practices for teaching miswak. These areas are closely aligned with the study's research questions and allowed participants to provide reflections, comments, and suggestions.

The topics explored were derived from previous literature^1,2,15,16^ and guided by the study's objectives. As recommended by Ritchie et al.,^17^ this approach ensured that only relevant aspects of miswak practice were included to build a comprehensive understanding of participants’ experiences.^17-19^ The topic guide was piloted before the actual FGDs to ensure its clarity, relevance, and alignment with both the research aims and the local context. It was also reviewed and validated by two experts in dental public health and qualitative research to ensure its relevance, clarity, and alignment with the study objectives before the focus group discussions were conducted.

During the FGDs, emerging themes, particularly those that were conflicting or underdeveloped in earlier discussions, were reviewed and validated in subsequent sessions, while the core topic areas remained unchanged. Given the participants’ diverse social and geographical backgrounds, some responded more actively to topics that aligned closely with their personal experiences and prior exposure to miswak in both daily practice and teaching.

### Field work

Before the interview sessions, invitation letters, attached with a copy of the study's ethical approval, were distributed to all dental educators in IIUM, informing them of the study's purpose. This initial letter was followed by an email containing an information sheet and a consent form. A reminder email was sent after seven days, and if there was still no response after an additional seven days, participants were contacted via phone. Once consent was obtained, interview appointments were scheduled at mutually agreed-upon dates and times.

The FGDs were conducted in English, as all participants are proficient in the language. Sessions took place in a seminar room within the educators’ workplace, selected jointly by the researcher and participants. Each session was expected to last approximately one hour, in line with standard durations for qualitative research interviews.^18^ The purpose of the study was explained beforehand, and participants were allowed to submit written questions and seek clarification before providing their informed consent.

With participants’ permission, discussions were audio-recorded using a discreet, high-quality recording device. Field notes were also taken during the sessions. All recordings were transcribed by a professional transcriber. To ensure accuracy, the researcher, who also conducted the interviews, reviewed the audio recordings and verified the transcripts. This step was particularly important as the transcriber encountered difficulty in understanding the accents of some international participants, resulting in initial transcription gaps. Participants were offered the opportunity to review their transcripts for any potentially sensitive content; however, none requested to do so. All transcripts were anonymised using coded identifiers (e.g., R1) to protect participants’ confidentiality.

### Qualitative data analysis

The interviews were audio-recorded, transcribed, and analyzed using the framework analysis method, which is commonly used in applied qualitative health research to organize and interpret thematic data. The analysis followed several stages, which include familiarization, identifying a thematic framework, indexing, charting, and mapping and interpretation.^17^

Initially, the transcripts were read repeatedly to achieve familiarization with the data. Two researchers independently reviewed the transcripts and developed preliminary codes reflecting recurring concepts and ideas expressed by participants. The coding approach was primarily inductive, allowing themes to emerge from the data while being informed by the study objectives and existing literature, reflecting a hybrid analytic approach. These initial codes were compared and discussed to develop a coding framework. The agreed coding framework was then applied systematically across all transcripts. Codes were subsequently grouped into categories and broader themes that reflected patterns within the data. Any discrepancies in coding or interpretation were resolved through discussion and consensus among the research team to ensure credibility and consistency of interpretation. Through this analytic process, four interrelated domains influencing miswak practice among dental educators were identified, namely self-care, conventional teaching, research evidence, and political/policy influences. These domains formed the conceptual basis for the model presented in Figure.

**Figure:**
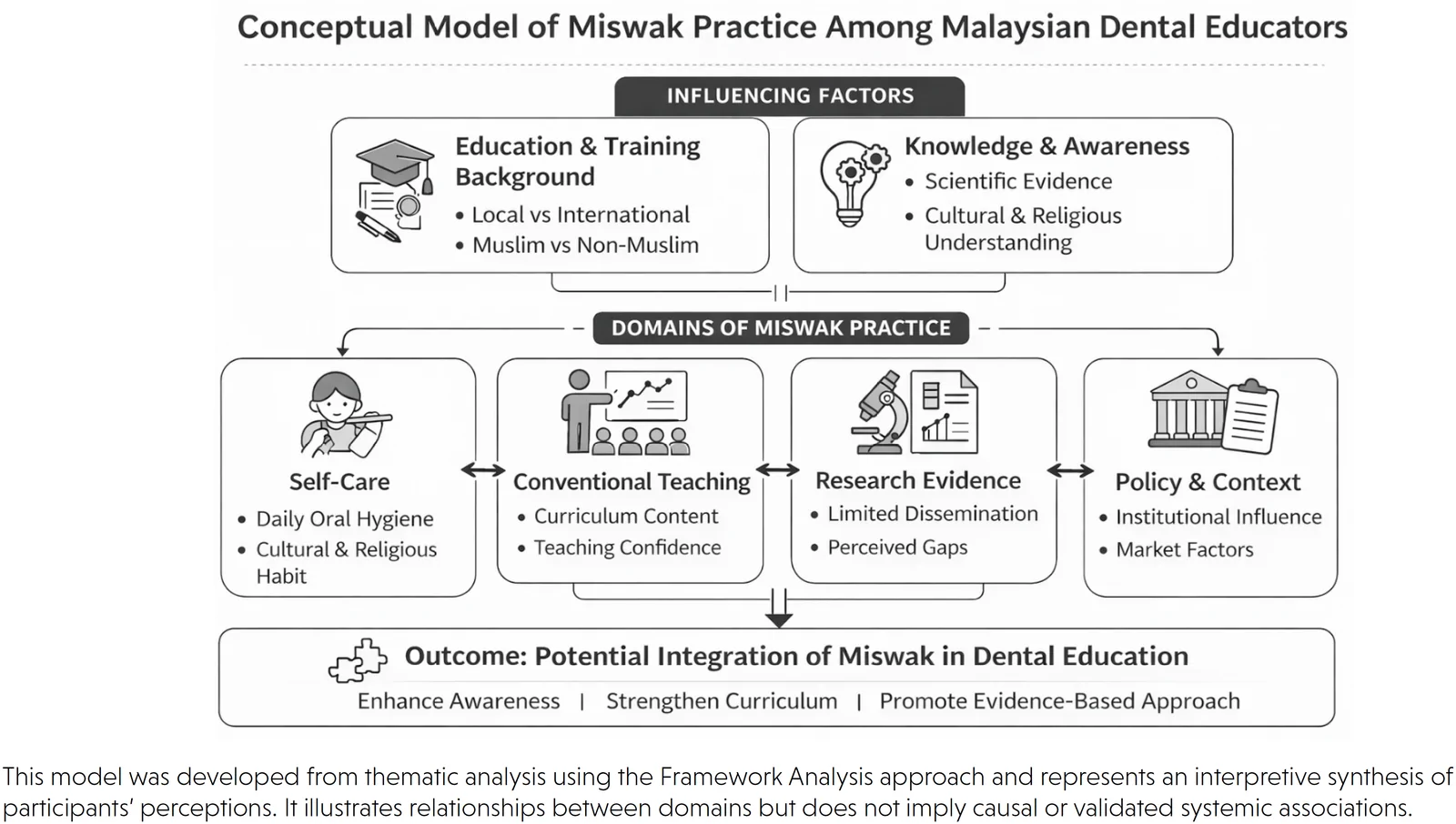
Interpretive conceptual model of domains influencing Miswak practice among Malaysian dental educators.

Data collection continued until thematic saturation was reached, defined as the point at which no new themes or concepts emerged from the discussions. After the fourth focus group discussion, the research team observed that similar issues and perspectives were repeatedly raised by participants, indicating that sufficient depth and coverage of the topic had been achieved. The focus group discussions were moderated by the first author (M.F.C.M.), a dental academic with training in dental public health and prior experience conducting qualitative research. The interviewer was familiar with the topic of miswak practice but did not hold a supervisory or evaluative role over the participants, which helped minimize potential power dynamics during the discussions.

### Researcher reflexivity

The primary interviewer was a dental academic familiar with the topic of miswak practice and working within the same academic environment as the participants. While this familiarity facilitated rapport and open discussion during the focus group sessions, steps were taken to minimize potential bias. A structured topic guide was used to ensure consistency across discussions, and participants were encouraged to express diverse viewpoints freely. During the analysis phase, interpretations of the data were discussed among members of the research team to ensure that the themes reflected the participants’ perspectives rather than the researchers’ assumptions.

### Trustworthiness of the data

To enhance the trustworthiness of the findings, several strategies were employed. Credibility was supported through prolonged engagement with the data, repeated reading of the transcripts, and discussions among members of the research team during the coding and theme development process. Dependability was strengthened by maintaining a clear audit trail of the analytical decisions made throughout the study. Confirmability was addressed through independent coding by two researchers and resolution of discrepancies through discussion and consensus. Transferability was facilitated by providing detailed descriptions of the study context, participant characteristics, and illustrative quotations from the focus group discussions.

## Results

Eleven respondents participated in four focus group discussions (FGDs) and they were grouped according to their backgrounds: (i) local Muslim (n=5); (ii) local non-Muslim (n=2); (iii) international Muslim (n=2); and (iv) international non-Muslim (n=2). The majority were female (54%; n = 6), and 30% (n = 3) had over 20 years of professional experience. All participants were Asian, held postgraduate qualifications, and were primarily trained in Western educational systems, with experience working in two or more organizations or sectors.

The conceptual model presented in Figure emerged from the thematic analysis conducted using the framework analysis approach. Recurring codes were grouped into categories that reflected four interrelated domains shaping miswak practice among dental educators, namely self-care, conventional teaching, research evidence, and political/policy influences. These domains interact dynamically and collectively shape how miswak is perceived and integrated within dental education and practice.

The following sections present the findings for each domain, supported by illustrative quotations from participants, which collectively informed the development of the conceptual model shown in Figure.

### Domain 1: Self-Care- Religious Tradition Meets Evidence

Many educators explained that miswak use is deeply embedded in religious and cultural traditions, often performed to fulfil Islamic obligations. Both local and international Muslim educators highlighted the religious significance of miswak. As one participant explained:

*"It is sunnah (religious practice)." FG3: (R2) 5 [international,* ♂*]*

Another participant described the longstanding cultural practice of using miswak for oral hygiene:

*"A long time ago, it has been used in our culture to clean the teeth." FG4: (R1) 2 [international, (*♂*)]*

This domain reflects how cultural and religious identity serves as a primary motivator for personal miswak use.

### Domain 2: Conventional Teaching- The Education Gap

Educators highlighted the critical role of evidence-based knowledge in shaping their teaching practice. Most expressed uncertainty and a lack of confidence in teaching miswak due to limited formal guidelines and scientific evidence:

*"I also think [there is a] lack of awareness or confidence [to use and teach] amongst dental educators because you know, for us, we learn from books and evidence." FG1: (R2) 7 [local* ♀*]*

*"No evidence. One thing is no evidence that miswak or this taboo has the same benefit of cleaning the tooth." FG3: (R2) 5 [international,* ♂*]*

Although many Muslim educators value the Sunnah, the limited rigorous evidence and absence of formal teaching frameworks resulted in minimal emphasis on miswak within dental curricula.

### Domain 3: Research Evidence Exists but Is Insufficiently Disseminated

The respondents recognized some local research supporting miswak's benefits, such as antibacterial and antiplaque properties, but expressed concerns about limited dissemination and international recognition:

*"I think the [one department at local university], they did something [research] on miswak, and that paper also reports the same [benefits of miswak]." FG1: (R4) 5 [local,* ♀*]*

*"But in terms of benefit like antibacterial properties, antiplaque and anti-gingivitis as discussed, the [paper however, is not being published well. We don't know whether the claiming is true or not." FG1: (R2) 5 [local,* ♀*]*

*"Miswak is not really well documented in terms of research. So, in my opinion, miswak itself is still lacking evidence, or the data is not published internationally." FG1: (R2) 7 [local,* ♀*]*

Participants expressed concerns that limited dissemination of research evidence may affect confidence in integrating miswak into dental teaching and practice.

### Domain 4: Political Policy - Market Forces and Institutional Resistance

Political and market influences were perceived as barriers to miswak's widespread acceptance and practice:

*"It's politics. They control the market; they don't want other new thing."*

*FG1: (R2) 7 [local,* ♀*]*

The absence of official guidelines and political resistance were quoted as significant factors limiting miswak's presence in dental curricula and practice.

### Influential factors: evidence-based practice (EBP) culture versus cultural heritage

#### Education and training

Respondents noted the absence of formal curriculum guidelines on miswak use during their dental training:

*"In terms of curriculum during the study itself, there is no guideline in the book. So, we don't have that guideline (for teaching)." FG1: (R2) 7 [local,* ♀*]*

This education gap contributed to inconsistent teaching and practice.

#### Knowledge and awareness

The level of knowledge and awareness is a key determinant of miswak practice. Respondents acknowledged that existing evidence, although localized and limited, greatly impacted their confidence and willingness to incorporate miswak into practice and teaching:

*"I also think [there is a] lack of awareness or confidence [to use and teach] amongst dental educators because you know, for us, we learn from books and evidence." FG1: (R2) 7 [local,* ♀*]*

*I think for me, what will really keep this product off will be effectiveness, obviously, with the evidence behind it." FG2: (R1) 6 [local,* ♂*]*

### Balancing benefits and barriers to enhance miswak usage

Despite the challenges, the educators viewed miswak as a complementary oral hygiene tool rather than a replacement for modern methods. They emphasized the need for innovation and collaborative efforts to improve miswak's acceptability and usability:

*"It should be used as addition [tool]. They should modify it to make it comfortable." FG3: (R2)*

*2 [international,* ♂*]*

*"If it being developed by a good and great company…Like you change its appearance, I think more people will be more receptive." FG2: (R1)*

*6 [local,* ♂*]*

The findings suggest that dental educators with predominantly Western-trained backgrounds demonstrate varied perceptions of miswak practice across four interrelated domains, consisting of self-care, conventional teaching, evidence-based research, and political policy. Cultural and religious values, prior education, and levels of knowledge and awareness influence these perceptions. A prevailing emphasis on evidence-based practice leads to limited and inconsistent integration of miswak due to the lack of comprehensive, widely disseminated research and formal guidelines. Nonetheless, educators recognize the cultural importance of miswak and advocate for further research and innovation to balance its benefits and barriers, thereby promoting its acceptance as a complementary oral hygiene practice.

## Discussion

This study explored dental educators’ perceptions of miswak practice and identified four interrelated domains influencing its use, incorporating self-care, conventional teaching, research evidence, and political or policy context. These domains were shaped by educational background and training of educators, as well as their level of knowledge and awareness regarding miswak. This study extends existing qualitative research by synthesizing educators’ perspectives into an interpretive conceptual model that highlights the interaction between cultural, educational, and evidence-based factors influencing miswak practice. Unlike previous studies that focus primarily on knowledge or attitudes, this study provides an integrated framework that may inform curriculum development and policy considerations in culturally diverse dental education settings.

Participants described miswak primarily as a culturally and religiously embedded oral hygiene practice, particularly within Islamic traditions. The use of miswak as part of daily self-care reflects a longstanding practice that predates modern toothbrushes and continues to be encouraged in religious teachings.^20^ Previous studies have also highlighted the cultural and religious significance of miswak in oral hygiene practices within Muslim communities.^21,22^ At the same time, systematic reviews and clinical studies have demonstrated that miswak possesses antimicrobial and antiplaque properties and can be comparable to conventional toothbrushes in reducing plaque and gingivitis adjunctively.^11,22^ These findings suggest that miswak represents a traditional practice that may also have scientifically supported oral health benefits.

Despite this potential, participants reported limited integration of miswak within dental teaching. Many educators expressed uncertainty about teaching miswak due to the perceived absence of structured curriculum guidelines and the limited emphasis on miswak during their own professional training.^5^ Similar observations have been reported in previous studies, where dental professionals trained within Western-oriented educational systems tended to prioritize conventional oral hygiene tools supported by widely disseminated scientific evidence.^5^ This highlights the role of curriculum development in shaping educators’ confidence and willingness to introduce traditional practices within dental education.^23^

Another key finding relates to the perceived limited dissemination of miswak-related research. Although several systematic reviews and clinical studies have demonstrated the oral health benefits of miswak,^6,9,11^ participants indicated that such evidence is not widely recognized within mainstream dental education. This perception may reflect the concentration of miswak research within specific regional or ethnopharmacological literature that may not be consistently incorporated into international dental education resources.^24^ Increasing the visibility of such evidence within dental curricula and professional education may therefore contribute to greater awareness and acceptance.^25^ This apparent discrepancy between the availability of scientific evidence and educators’ perceptions may reflect gaps in knowledge translation rather than a true absence of evidence. It suggests that the issue lies not only in evidence generation but also in how such evidence is disseminated, contextualized, and incorporated into dental curricula. This underscores the importance of strengthening knowledge translation strategies within dental education to bridge the gap between research and practice.

Participants also perceived broader contextual influences, including institutional and market factors, that may affect the adoption of alternative oral hygiene tools such as miswak. While these views reflect the perceptions of participants rather than verified structural barriers, they suggest that broader professional and policy contexts may influence how traditional oral health practices are positioned within modern dental education.^26^ Importantly, participants did not view miswak as a replacement for conventional oral hygiene tools but rather as a potential complementary practice.^5^ This perspective is consistent with clinical evidence suggesting that miswak may provide additional benefits when used alongside modern toothbrushes.^11^ However, proper instruction on technique and usage is essential, as incorrect use may reduce its effectiveness.^27^ Product innovation and improved design may also enhance its acceptability and usability among modern users.^28^

The findings of this study should be interpreted in light of its methodological context. The qualitative focus group approach allowed an in-depth exploration of educators’ perceptions and experiences. By grouping participants with similar socio-cultural backgrounds, the study facilitated open discussion on potentially sensitive topics. The use of open-ended questions enabled participants to express their views freely and provided rich contextual data regarding miswak practice.^29^ Nevertheless, as with most qualitative studies, the findings reflect participants’ perspectives within a specific institutional context and time.^30^ Perceptions and practices may evolve as new evidence emerges and as awareness of miswak increases within professional communities.^31^

Future research involving larger multi-institutional and multicultural samples, as well as mixed-methods approaches, may provide broader insight into how traditional oral hygiene practices such as miswak can be appropriately integrated into contemporary dental curricula and public health strategies.^32,33^ Additional studies exploring students’ perceptions, learning experiences, and educational outcomes related to miswak teaching may also strengthen the evidence base in this field. Overall, this study highlights the complex interaction between cultural heritage, scientific evidence, and professional education in shaping attitudes toward traditional oral health practices such as miswak.

## Conclusion

This qualitative study identified four interrelated domains influencing miswak practice among dental educators: self-care, conventional teaching, research evidence, and political or policy context. The findings suggest that educators’ education and training background, along with their level of knowledge and awareness, shape perceptions of miswak, and influence its integration within dental education and practice. While miswak is widely recognized for its cultural and potential oral health benefits, its incorporation into dental teaching remains limited. Strengthening research dissemination, developing educational guidance, and promoting culturally sensitive approaches may support the appropriate integration of miswak as a complementary oral hygiene practice within contemporary dental education.

## Data Availability

The datasets generated during and/or analyzed during the current study are available from the corresponding author on reasonable request.
